# Tissue-specific patterns of gene expression in the epithelium and stroma of normal colon in healthy individuals in an aspirin intervention trial

**DOI:** 10.1186/s12881-015-0161-6

**Published:** 2015-03-24

**Authors:** Sushma S Thomas, Karen W Makar, Lin Li, Yingye Zheng, Peiying Yang, Lisa Levy, Rebecca Yvonne Rudolph, Paul D Lampe, Min Yan, Sanford D Markowitz, Jeannette Bigler, Johanna W Lampe, John D Potter

**Affiliations:** Fred Hutchinson Cancer Research Center, Seattle, WA 98109 USA; M.D. Anderson Cancer Center, Houston, TX 77030 USA; Case Western Reserve University School of Medicine, Cincinnati, OH 44106 USA; Amgen Corporation, Seattle, WA 98119 USA

**Keywords:** Gene expression, Colon stroma, Colon epithelium, Microarray, Colon biopsy, UGT1A6, Aspirin

## Abstract

**Background:**

Regular aspirin use reduces colon adenoma and carcinoma incidence. UDP-glucuronosyltransferases (UGT) are involved in aspirin metabolism and clearance, and variant alleles in *UGT1A6* have been shown to alter salicylic acid metabolism and risk of colon neoplasia.

**Methods:**

In a randomized, cross-over, placebo-controlled trial of 44 healthy men and women, homozygous for UGT1A6*1 or UGT1A6*2, we explored differences between global epithelial and stromal expression, using Affymetrix U133 + 2.0 microarrays and tested effects of 60-day aspirin supplementation (325 mg/d) on epithelial and stromal gene expression and colon prostaglandin E2 (PGE2) levels.

**Results:**

No statistically significant differences in gene expression were observed in response to aspirin or *UGT1A6* genotype, but tissue PGE2 levels were lower with aspirin compared to placebo (p <0.001). Transcripts differentially expressed between epithelium and stroma (N = 4916, P <0.01, false discovery rate <0.001), included a high proportion of genes involved in cell signaling, cellular movement, and cancer. Genes preferentially expressed in epithelium were involved in drug and xenobiotic metabolism, fatty acid and lipid metabolism, apoptosis signaling, and ion transport. Genes preferentially expressed in stroma included those involved in inflammation, cellular adhesion, and extracellular matrix production. Wnt-Tcf4 pathway genes were expressed in both epithelium and stroma but differed by subcellular location.

**Conclusions:**

These results suggest that, in healthy individuals, subtle effects of aspirin on gene expression in normal colon tissue are likely overwhelmed by inter-individual variability in microarray analyses. Differential expression of critical genes between colonic epithelium and stroma suggest that these tissue types need to be considered separately.

**Electronic supplementary material:**

The online version of this article (doi:10.1186/s12881-015-0161-6) contains supplementary material, which is available to authorized users.

## Background

Regular aspirin (acetylsalicylic acid) use reduces risk of colorectal cancer (CRC) mortality as well as incidence of CRC and adenomas [1-4]. Aspirin is hypothesized to exert its antineoplastic effects via several mechanisms. It may reduce risk of neoplasia by blocking synthesis of prostaglandin E_2_ (PGE_2_) through irreversible acetylation and by competitively inhibiting cyclooxygenase (COX)-1 and COX-2 [5] Aspirin may also influence risk through COX-independent pathways, such as the DNA mismatch repair system, epithelial cell proliferation and apoptosis, and antioxidant enzymes [6]. Aspirin reduces risk of sporadic colorectal adenomas within a few years but requires up to a decade to reduce risk of invasive cancer, suggesting that the effects of aspirin occur early in carcinogenesis or may even influence susceptibility of normal colonic tissue [4].

Aspirin treatment of colon cancer cell lines results in numerous changes in gene expression [7]. However, gene expression differences exist between colorectal tumor and normal epithelium [8-12] and colorectal tumor endothelial cells maintained in culture do not completely recapitulate expression patterns observed *in vivo* [13]. Therefore, it is likely that complex interactions between epithelium and stroma may be missed in simplified *in vitro* models of colon biology.

Previous studies designed to characterize the separate contributions of colonic stroma and colonic epithelium to carcinogenesis have focused on comparisons of tumor stroma to normal stroma, or tumor tissue to paired surrounding normal tissue [11,12,14,15]. Because these gene expression studies examined biopsy specimens from intact colon that were not dissected before analysis, the results were confounded by the contributions of multiple and heterogeneous cell types to overall expression signatures. More recently, a molecular pathway-based approach has been used to analyze the changes that occur during tumorigenesis [8]. Mojica and Hawthorn [16] have presented a data set of gene expression in normal colonic epithelial cells and compared them to a publicly available data set of tumor and matched normal colon data. However, to date, there has been no large-scale attempt to compare gene expression in colonic epithelium from normal healthy individuals to that in stroma from the same individuals or to evaluate the effects of potential preventive treatments on these different tissues.

Acetylsalicylic acid is rapidly deacetylated to salicylic acid, which is then further metabolized [17]. Glucuronidation of salicylic acid is an important pathway for elimination of the drug [17]. UGT1A6, a polymorphic UDP-glucuronosyltransferase (UGT), can conjugate salicylic acid [18]. Several studies suggest that the protein product of the *UGT1A6* variant allele has altered enzyme activity, which, in turn, affects aspirin metabolism [19-21]. Modifying effects of UGT1A6 genotype have been reported in some studies of adenomas [22,23] but not others [24], and not in studies of colon cancer [25,26]. Independent of aspirin use, the *UGT1A6* variants have been reported to influence adenoma recurrence [27] and CRC risk [28].

The objectives of this study were to measure effects of an aspirin intervention on gene expression in normal colonic epithelial and stromal tissue in healthy humans and to determine whether response differed by UGT1A6*2 genotype. We also sought to characterize gene expression differences within colonic tissue microenvironments by identifying genes that were differentially expressed between epithelial and stromal tissue.

## Methods

### Ethics statement

All study procedures and materials were approved by the Fred Hutchinson Cancer Research Center Human Research Protection Program, Institutional Review Board Committee C and informed, written consent was obtained from all participants prior to their starting the study.

### Participants

**Figure 1:**
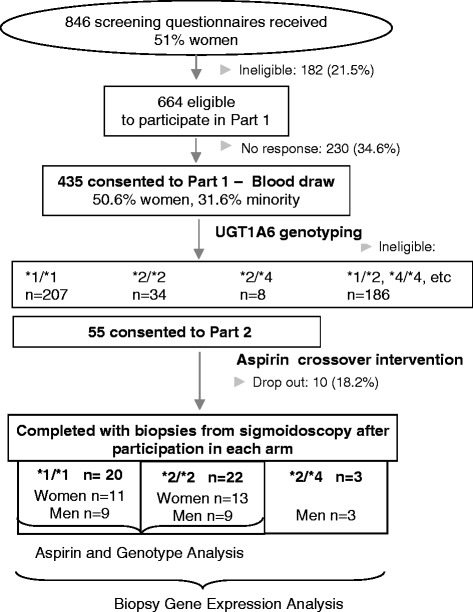
**Flow chart of participant enrollment and study design.**

We recruited healthy men and women, ages 20 to 45 y, from the greater Seattle area between June 2003 and March 2007. Participants were recruited from among those who completed a cross-sectional study of diet and aspirin metabolism (Figure 1). Potential eligibility was assessed by questionnaire. Exclusion criteria included tobacco use, consumption of >2 alcoholic beverages/d (equivalent to 720 ml beer, 240 ml wine, 90 ml hard liquor), regular use of prescription or over-the-counter medications, known intolerance of aspirin or other non-steroidal anti-inflammatory drugs (NSAID), weight loss or gain of >4.5 kg in the past year, current or planned pregnancy, breastfeeding, bleeding disorder, anemia, renal insufficiency, hepatic dysfunction (e.g., cirrhosis, hepatitis, abnormal liver function tests), chronic lung disease, hypertension, congestive heart failure, angina, recent myocardial infarction, history of endocarditis, aortic or iliac aneurysm, history of stroke or transient ischemic attack, diabetes, recent pelvic surgery, history of gastrointestinal disorder (e.g., gastric or duodenal ulcer, ulcerative colitis, Crohn disease, celiac sprue, HNPCC, familial adenomatous polyposis, pancreatic disease, previous gastrointestinal resection, radiation or chemotherapy) and cancer (other than non-melanoma skin cancer).

As part of the cross-sectional study, participants completed a self-administered food frequency questionnaire and a health and demographic survey, and provided a fasting morning blood that was used for *UGT1A6* genotyping [29]. We determined the UGT1A6*2 genotype of 434 participants by Sanger sequencing of a 268 bp fragment of exon 1, amplified by PCR as described previously [30].

### Study design

The study was a randomized, double-blind crossover clinical trial designed to examine the effects of aspirin on gene expression in colonic mucosa. Our goal was to have at least 40 participants complete both arms of the trial. Participants were selected on the basis of their genotype of rs2070959 (T181A) and rs1105879 (R184S) -- 20 with the UGT1A6 *1/*1 genotype (homozygous for the major allele at both SNPs) and 20 with the UGT1A6 *2/*2 genotype (homozygous for the minor allele at both SNPs). All participants with a *2/*2 genotype and sex-matched participants with a *1/*1 genotype were invited to consider participation in the trial. Additionally, 3 participants with the *2/*4 (T181A + R184S/R184S only) genotype were randomized.

To ensure that participants could take aspirin safely for 60 d and tolerate sigmoidoscopy, each participant underwent a clinical assessment before entering the study that included a detailed medical history, measurement of blood pressure and complete blood count, liver panel, chemistry panel, blood urea nitrogen, serum creatinine, urinalysis, and in women only, a pregnancy test. In addition, a physician interviewed and examined participants before each of their sigmoidoscopies. The laboratory assays to assess the health of participants were completed by a commercial lab (CLIA licensed Quest Diagnostics, Seattle, WA).

Because this was a randomized crossover trial, each person served as his/her own control, receiving both intervention (aspirin) and control (placebo) at 2 different periods, with a washout period between periods. Participants were randomly assigned, blocked on sex and genotype, to the order in which they received aspirin or placebo. Eligible participants took 325 mg aspirin or a visually identical placebo by mouth daily, for 60 d. The washout period between intervention periods was 3 mo. A sigmoidoscopy was performed at least 60 d after day 1 of each intervention period. Participants took the study medication up until 24 h before their sigmoidoscopies. During each treatment period, we monitored adherence to the study medication by pill count.

### Sigmoidoscopy, biopsy collection, and tissue separation

Participants prepared for sigmoidoscopy by following standard instructions for use of Fleet Phospho-soda Oral Saline Laxative (Lynchburg, VA), which included adherence to a clear-liquid diet for 24 h before the procedure. At each sigmoidoscopy, we used large-cup flexible biopsy forceps (Precisor Disposable, Bard, Billerica, MA) to obtain biopsies of normal-appearing mucosa from the sigmoid colon. Forty-five participants completed both sigmoidoscopies. Within 1 min of removal from the colon, three biopsies were transferred to Hank’s buffer containing 20 mM EDTA and 40 mM dithiothreitol at 4°C and were held on ice for 10–15 min. The epithelial cells were separated from the stromal layer by vortexing [31]. The stromal layer was removed with an autoclaved toothpick and epithelial fraction was then collected by centrifugation. Both fractions were frozen and stored in liquid nitrogen until RNA extraction. Additionally, two biopsies were kept intact and frozen and stored in liquid nitrogen.

### RNA extraction and microarray procedures

Each participant’s epithelial and stromal samples were processed at the same time. Total RNA was extracted with RNeasy extraction kits (Qiagen, Valencia, California) and treated with DNAse I according to the manufacturer’s protocol. The integrity of total RNA samples was verified by visual analysis of 18S and 28S bands on an ethidium bromide-stained 1.5% agarose gel. RNA was quantified using the Quant-iT RiboGreen RNA assay Kit (Molecular Probes, Eugene, Oregon). We obtained sufficient RNA from 44 of the 45 participants. This resulted in obtaining total RNA from 88 epithelial samples and 88 matched stromal samples. cRNA was synthesized from 100 ng of total RNA, biotin-labeled by the GeneChip Two-cycle cDNA synthesis labeling protocol (Affymetrix, Inc., Santa Clara, California), and then hybridized to HGU133 plus 2.0 expression arrays. Each microarray contained 54,675 probes representing 38,500 genes. The quality control (QC) criteria used to assess sample and chip performance were fold-amplification after two cycles of *in vitro* transcription (to be at least 40-fold), and increasing signal strengths and ‘present’ calls for poly-A RNA spike-in controls (lys, phe, thr, dap) and hybridization controls (bioB, bioC, bioD and cre). Microarray data are publicly available in Gene Expression Omnibus (GEO) [32].

### Real-time quantitative reverse transcription-polymerase chain reaction (qRT-PCR)

To validate the microarray data, Taqman RT-PCR was performed on a total of 20 RNA samples, which included both pairs of stromal and epithelial samples from 10 of the 44 individuals. To ensure an RT performance of equal quality, all samples were reverse transcribed simultaneously by a two-step reverse transcription with SuperScript RT II (Invitrogen, Carlsbad, California). Relative gene expression was examined by Taqman PCR using 2X Taqman Gene Expression Master Mix (Applied Biosystems, Foster City, California) and Taqman Gene Expression Assays for two epithelial genes (ABCC3, MLPH; assay identifiers Hs00358677_m1 and Hs00983106_m1) and two stromal genes (ITGα8 and ANXA1; assay identifiers Hs00943530_m1 and Hs00945401_m1). Human β-glucuronidase (GUSB) was used as the normalization control based on its consistent expression across all samples in the microarray analysis. Relative quantification was performed with the ABI Prism 7900 HT sequence detection system and calculated by the comparative C_T_ method.

### PGE_2_ analysis

PGE_2_ in whole biopsies collected at the end of each treatment period was measured with enzyme immunoassay kits from Assay Design (Farmingdale, NY). Briefly, each frozen biopsy tissue sample was transferred to a sealed microcentrifuge tube to which 500 μl of ice-cold tissue homogenization buffer had been added previously [33]. Each sample was homogenized by an Ultrasonic Processor (Misonix, Farmingdale, NJ) at 4°C for 3.5 min × 2 with a 1-min rest in between and then centrifuged at 16,000 rpm for 5 minutes at 4°C. An aliquot (100 μl) was mixed with 100 μl of assay buffer and acidified with 20 μl 1 N citric acid. The acidified solution was then applied to a Sep-Pak C18 cartridge (Waters Corp., Milford, MA) that had been preconditioned with methanol and water. Prostaglandins were eluted with 2 ml of hexane:ethyl acetate (1:1). The eluate was evaporated under a stream of nitrogen, and the residue was dissolved in 25 μl ethanol and 200 μl assay buffer. PGE_2_ was then measured with ELISA kits according to the manufacturer’s instructions. Protein levels were determined by a Bradford protein assay (Bio-Rad, Hercules, CA). Levels of PGE_2_ were normalized to protein concentrations and expressed as ng/mg protein.

### Data analysis

#### Microarray analysis

Data were normalized by the Robust Multi-array Analysis with correction for GC content of the oligo (GC-RMA) [34]. Subsequently, the probe-set expression levels were log2-transformed. Nonspecific filtering was done where estimated intensities were required to be above 100 fluorescence units in at least 25% of the samples and the interquartile range (IQR) across all of the samples on the log base 2 scale was expected to be at least 0.6. A total of 6227 genes passed nonspecific filtering. Differential expression between tissue types in biopsies collected at the end of each treatment period was determined by using a paired t-test. The false discovery rate (FDR) was calculated using the procedure proposed by Benjamini and Hochberg [35]. Visualization of the statistically significant genes was performed in GeneSpring 7.0 with per chip and per gene normalizations (Agilent Technologies, Wilmington, Delaware). Ingenuity Pathways Analysis (IPA, Release Summer 2013) (Ingenuity Systems, Redwood City, California, http://www.ingenuity.com) was used to determine the relevant functions and pathways expressed in each tissue type. A list of the top genes in each pathway was identified using GeneSpring and heat maps for each pathway were generated by clustering these genes in Gene Cluster 3.0 [36] and then visualizing the clusters using Java TreeView [37]. Differential expression between interventions within each tissue type and between two *UGT1A6* genotypes (*1/*1 and *2/*2) were also examined by using the same paired t-test and the generalized estimating equation approach. Genes that were statistically significantly differentially expressed between stroma and epithelium at the end of both treatment periods were included in further pathway analysis.

#### PGE_2_ analysis

Because the distributions of PGE_2_ levels measured in biopsies collected at the end of each treatment period were skewed, the data were logarithmically transformed. The difference between treatments was determined by using paired t-test and a random-effects model that takes into account the crossover design. The comparison between aspirin and placebo was further examined after stratification for sex, age, and *UGT1A6* genotypes.

## Results

Fifty-five individuals (23 men), mean (SD) age 30.1 (6.5) y, were randomized. Ten participants discontinued the study: 3 did not tolerate the Fleets Phospho-soda preparation prior to the first sigmoidoscopy, 2 started on prescription medications, 2 refused sigmoidoscopy, 3 left for reasons unrelated to the study and sufficient total RNA could not be extracted from one participant. Forty-two participants were included in the analysis of intervention and UGT1A6 genotype effects (Table 1) and an additional 3 participants with the UGT1A6 *2/*4 genotype were included in the expression array analysis of stromal and epithelial tissue. (Initially, we randomized *2/*4 individuals into the study because the phenotype was thought to be similar to that of *2/*2; however, with the emergence of new data [38], we decided that the 2 genotypes may be sufficiently different and therefore excluded *2/*4 individuals from the intervention analysis).

**Table 1:** **Characteristics of participants included in the analysis of the effects of aspirin and** ***UGT1A6*** **genotype on gene expression in colonic epithelium and stroma**

	UGT1A6*1/*1		UGT1A6*2/*2	
	**Men n = 9**	**Women n = 11**	**Men n = 9**	**Women n = 13**
Age, years (SD)	30.0 (4.65)	30.9 (5.8)	30.2 (5.6)	29.7 (7.8)
Height, cm (SD)	180.9 (5.6)	168.5 (7.2)	179.0 (7.8)	163.2 (8.0)
Weight, kg (SD)	83.6 (8.4)	69.0 (12.0)	88.2 (26.5)	71.3 (15.5)
BMI, kg/m^2^ (SD)	25.6 (2.6)	24.2 (3.2)	27.5 (7.8)	26.7 (5.2)
Minority, n (%)	2 (22.2)	3 (27.3)	3 (33.3)	2 (15.4)
Race *n* (%)				
Caucasian	6 (66.7)	6 (54.6)	6 (66.7)	11 (84.6)
Asian	1 (11.1)	1 (9.1)	2 (22.2)	1 (7.7)
African-American	0	1 (9.1)	0	0
Other	2 (22.2)	3 (27.2)	1 (11.1)	1 (7.7)
Family history of CRC, n (%)	0 (0.0)	1 (9.1)	1 (11.1)	0 (0.0)
Take supplements, n (%)	3 (33.3)	2 (18.2)	4 (44.4)	6 (46.2)
Ever smoked n (%)	1 (11.1)	3 (27.3)	1 (11.1)	1 (7.7)
Energy, kcal/d (SD)	1495 (373)	1468 (474)	1770 (459)	1505 (361)
Fruit, servings/d (SD)	1.3 (1.0)	1.9 (0.9)	2.5 (1.8)	2.9 (2.3)
Vegetables, servings/d (SD)	1.5 (1.2)	2.3 (1.2)	1.7 (1.0)	2.6 (2.1)
Caffeine mg/d (SD)	68 (92)	76 (60)	125 (147)	118 (71)
Calcium mg/d (SD)	926 (508)	760 (373)	782 (267)	773 (406)
Fat g/d (SD)	57 (20)	56 (20)	67 (29)	46 (20)
Dietary fiber, g/d	15.9 (8.3)	15.5 (5.6)	19.2 (9.0)	20.4 (8.4)

Abbreviations: CRC, colorectal cancer; SD, standard deviation.

Asterisks are used as part of the specific classification of the UGT1A6 genotype.

### Tissue-specific patterns of gene expression in epithelium and stroma of normal human colon

From 38,500 genes analyzed by microarray, we identified genes that were statistically significantly differentially expressed between epithelium and stroma in each treatment period. Because there were no intervention differences (see below), we combined the observations and restricted subsequent analyses to those genes that were differentially expressed in both treatment periods (P <0.01, FDR <0.001). This resulted in further exploration of 4916 genes. Of these, 2088 were higher in stroma and 2828 were higher in epithelium. The entire list of statistically significant genes and their expression values is available in Additional file 1.

**Figure 2:**
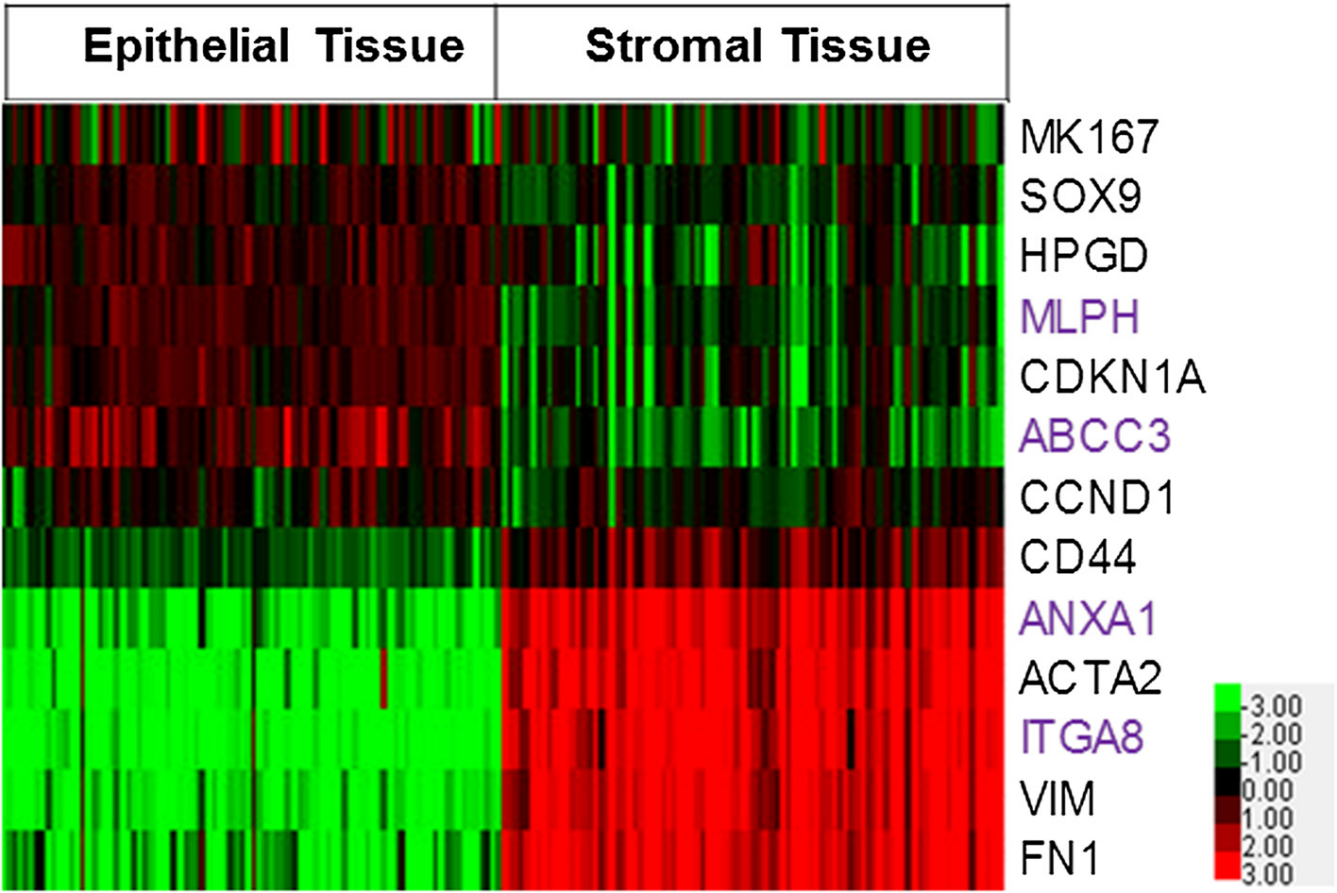
**Gene expression of known stromal and epithelial genes.** Java Treeview heatmap of normalized relative gene expression (log 2 intensities) for genes whose expression is known to be restricted to epithelium or stroma from previous immunohistochemistry studies (black text). Genes analyzed by qRT-PCR in Figure 3 are also shown (purple text). Red indicates over-expressed genes and green indicates under-expressed genes; and the expression level is proportional to the brightness of the color (see color bar). Black indicates no difference in expression level between the two tissue types and lighter coloring indicates lower overall expression.

**Figure 3:**
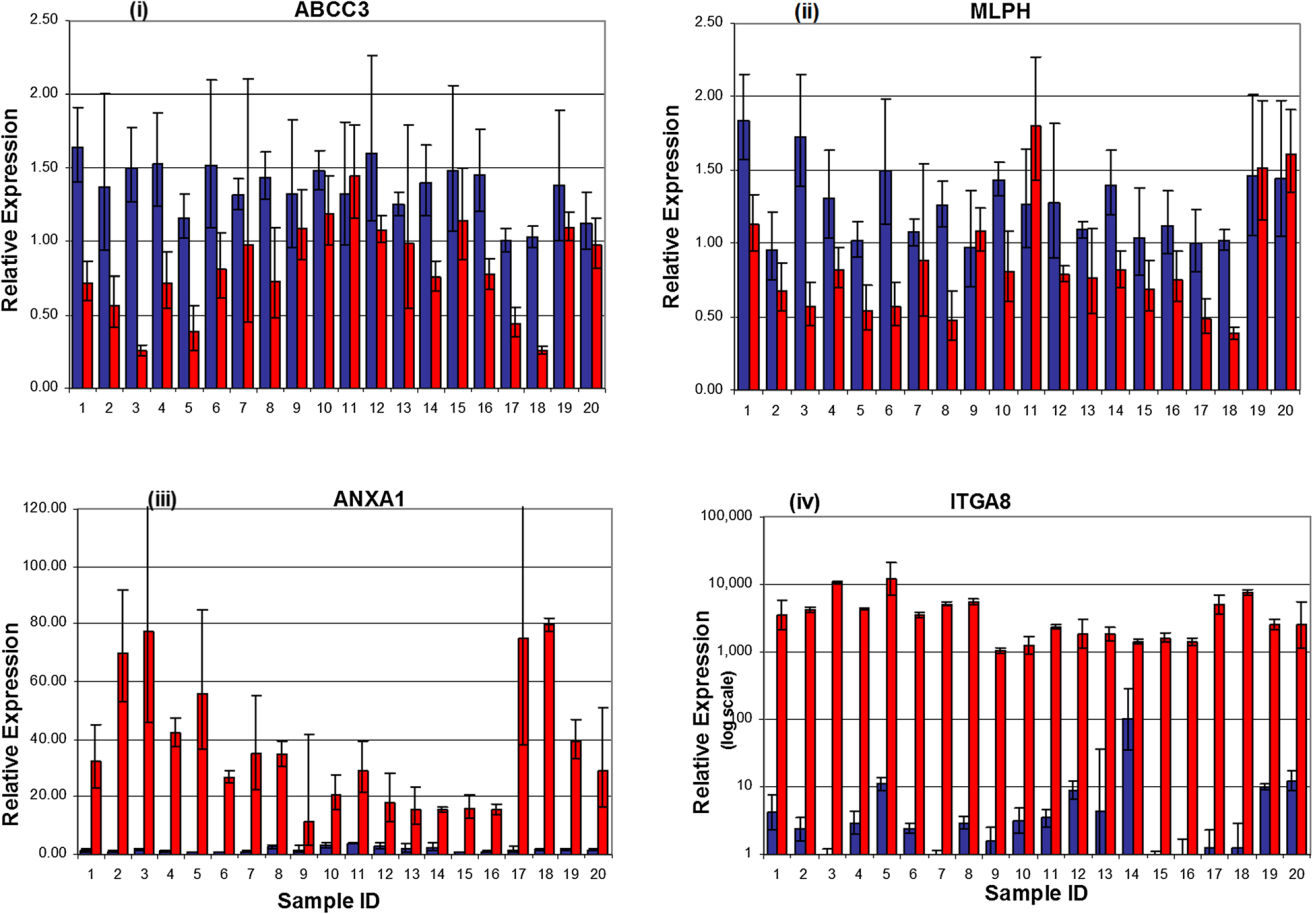
**Real-Time PCR confirms differences in gene expression in colonic epithelium and stroma.** Quantitative real-time RT-PCR was done on both biopsies (taken at two separate visits) from 10 study participants for four candidate genes. The histogram bars from the same individual are located next to each other, i.e., samples 1 and 2 are from the same person but taken at two separate visits. Total RNA was reverse transcribed and then amplified by PCR and normalized to β-glucuronidase (GUSB) expression. Blue bars -epithelial tissue. Red bars -stromal tissue. Upper panel shows expression of **(i)** ABCC3, an epithelial gene involved in drug and lipid metabolism and **(ii)** MLPH (melanophilin), an epithelial gene involved in protein binding and protein transport. Lower panel shows expression of **(iii)** ANXA1 (annexin A1), a stromal gene involved in cell migration and cell adhesion and **(iv)** ITGA8 (integrin α8), a stromal gene involved in extracellular matrix formation.

Separation of stromal and epithelial layers was verified by examining genes that are known to be expressed in epithelium or stroma from immunohistochemistry staining in multiple studies (Figure 2). Expression of α-smooth muscle actin (ACTA2), vimentin (VIM), and fibronectin (FN1) was restricted to the stromal compartment, which is consistent with the known expression of these genes specifically in pericryptal myofibroblasts [39-42]. Conversely, expression of cyclin D1 (CCND1), p21 (CDKN1A), and 15-PGDH (HPGD) has been observed consistently in the top portion of the crypts [11,43-47] and this correlated with the higher expression observed in our epithelial samples. For genes known to be expressed in the crypt base such as CD44, SOX9, and Ki-67 [44,47-51], our data revealed that SOX9 and Ki-67 (MKI67) showed higher expression in the epithelial fraction, whereas CD44 showed higher expression in the stromal fraction. Stromal and epithelial patterns of expression observed in the microarray data were confirmed by qRT-PCR of selected genes (Figure 3). Expression of the selected epithelial-specific genes, ABCC3 and MLPH1, was detectable in the stromal samples, but at a lower level than in the epithelial samples. In contrast, the selected stromal-specific genes, ANXA1 and ITGA8, generally lacked detectable expression in the epithelial samples.

To understand the biologic processes represented by differentially expressed genes, we analyzed those showing statistically significant differences with Ingenuity Pathways Analysis (IPA). Although both compartments expressed genes involved with cancer, cell death, cell signaling and cellular movement, several pathways and biologic functions appeared to be unique to one or the other tissue. Table 2 lists the major differentially expressed pathways and functions by tissue type.

**Table 2:** **Statistically significant pathways and functions in colonic epithelium and stroma**

	**Epithelial genes**	**p-value**
Canonical pathways	Xenobiotic Metabolism signaling	0.000124
	Fatty acid Metabolism	0.00262
	*Apoptosis Signaling*	0.22
Biological function	Lipid Metabolism	6.25E-07-2.47E-02
	Small Molecule Biochemistry	6.25E-07-2.47E-02
	Molecular Transport	5.89E-05-2.47E-02
	*Carbohydrate Metabolism*	7.48E-04-2.47E-02
	*Drug Metabolism*	1.80E-03-2.47E-02
	*Protein trafficking*	2.34E-03-2.47E-02
	*Nucleic acid Metabolism*	8.58E-03-2.47E-02
	*Amino acid Metabolism*	1.23E-02-2.47E-02
	**Stromal genes**	**p-value**
Canonical pathways	*Complement System*	2.49E-08
	*Antigen Presentation Pathway*	0.00000018
	IL-4 signaling	0.0000656
Biological function	Inflammation	8.74E-28-8.17E-06
	Cell-to-cell signaling	1.90E-27-1.09E-05
	*Immunological Disease*	5.34E-25-4.44E-06
	Hematological System Development & Function	4.50E-19-1.12E-05
	Immune Response	4.50E-19-1.12E-05

Ingenuity Pathway Analysis of selected biological functional groups and metabolic and signaling pathways for all statistically significant genes of both tissue types. Range of p-values indicates the significance values of the specific sub-functions associated with that particular high-level function. *Italic font indicates function was unique to that particular tissue type and was essentially not expressed in the other tissue.*

#### Lipid metabolism and small molecule biochemistry

Lipid metabolism and small-molecule-biochemistry pathways were highly represented in epithelial tissue. Several of the genes highly expressed in epithelium were categorized as being involved in the accumulation, biosynthesis, metabolism, and modification of fatty acids and lipids. For example, ACADVL and DECR2 are involved in modification of lipids; CYP3A4 is involved in modification and beta-oxidation of fatty acids; FAAH and PPARA with catabolism and degradation of fatty acids; and CYP51A1 in the biosynthesis of cholesterol. Additional file 2 depicts the expression pattern in epithelium and stroma of the top 25 (determined by p-values associated with differential expression) genes in the category of lipid metabolism.

Within the category of lipid metabolism, the prostaglandin pathway was well represented. Expression of HPGD, PTGDR, and PTGES2 was higher in the epithelium; in contrast, expression of PTGS1 (COX-1), PGDS, and PTGER2 was higher in the stroma (see Additional file 3). The expression of COX-1 is constitutive in normal tissue unlike COX-2 (PTGS2), the expression of which is induced only in inflamed tissue or cancer [52]. COX-2 expression was not detected in either stroma or epithelium. HPGD is known to be highly expressed in normal colonic epithelial cells near the top of the crypt and has been shown to block the prostaglandin-synthesizing activity of COX-2 [43].

#### Molecular transport

Genes such as CLIC5, CLCN2 (chloride transport), KCNK5 (potassium channel family 5), SLC12A2 (K^+^/Cl^−^ transporter), SLC9A2 (sodium/hydrogen exchanger), SLC26A3 (chloride anion exchanger), and SCNN1B (involved in the flux of Na) (see Additional file 4) were all more highly expressed in the epithelial fraction. In contrast, the majority of stromal-specific molecular transport genes, such as VCAM1, CAV1, ITGB2, chemokine receptors and ligands, were in the categories of Ca^2+^ flux, quantity, and mobilization (data not shown).

#### Drug and xenobiotic metabolism signaling

Consistent with previously published data [9,10], our results showed that this critical pathway was highly expressed in the colonic epithelium; relevant genes included ABCC3, a member of the MRP family of multi-drug-resistance genes, UGT1A1, UGT1A6, GSTA1, GSTT1, GSTO2 (members of the Phase II conjugating enzyme family), MAOA (mono-amine oxidase A), CYP3A5 (involved in oxidation of testosterone), and members of the sulfotransferase family (SULT1A1, 1A2, 1A3 and 1B1) (see Additional file 5). Not all gene expression related to drug and xenobiotic-metabolism signaling was confined to the epithelium, however: members of the GST mu family (GSTM1, M2, and M3) were upregulated in the stroma as was MAOB, a gene known to have high activity in lymphocytes and platelets [53].

#### Inflammation and immune response

Genes involved in inflammation, angiogenesis, wound healing, and immune responses were highly expressed in the stromal compartment (see Additional file 6); these included genes encoding cytokine receptors (IL10RA), chemokines and chemokine receptors (CCL18, CCR2), inflammatory mediators (C4A), growth factors (CSF1), and adhesion proteins (PECAM1). This immune-related expression pattern represents the major canonical pathways unique to the stroma and reflects the presence of multiple cell types including lymphocytes, monocytes, and endothelial cells.

#### Extracellular matrix and cell adhesion

Matrix modeling and extracellular matrix (ECM) genes were expressed preferentially in the stroma. A search of IPA for “Extracellular Matrix” matched 29 functions with 399 associated molecules. Of these, 80 were expressed preferentially in the stroma and 40 were expressed preferentially in the epithelium. ECM genes, collagen types III and V (COL3A1 and COL5A1), decorin (DCN), dystrophin (DMD), lumican (LUM), and TIMP1 were expressed in stroma, along with laminins LAMA2, LAMA4, LAMB1 and LAMC1 (see Additional file 7). Laminins LAMA3 and LAMC2, had higher expression in the epithelial fraction, along with LAMA1. This is similar to the expression pattern seen in the small intestine [10], where the laminin A chain is predominantly associated with differentiated epithelial cells [54]. Henesin (DMBT1) showed higher expression in the epithelium, which is consistent with its role in the formation of columnar epithelium [10].

#### Wnt-β-catenin pathway

The Wnt-β-catenin pathway plays an important role in epithelial-stromal cell development and differentiation [55,56]. The roles of Wnt and β-catenin in colon carcinogenesis are well established [57,58]. Wnt pathway-related genes were identified from the Ingenuity database. Among the 4916 statistically significant genes, two distinct tissue-specific patterns of Wnt-related genes were observed, with 21 genes expressed more highly in the epithelium and 21 genes expressed more highly in the stroma (see Additional file 8). The genes preferentially expressed in epithelium encoded cytoplasmic Wnt pathway-associated proteins whereas secreted proteins (e.g., DKK3, Wnt5A, and Wnt5B) were expressed preferentially in stroma. Components of several other signaling pathways (MAPK, TGF-β, IL-1, IL-6) as well as tight-junction signaling, were represented in both epithelium and stroma (data not shown).

### Aspirin and *UGT1A6* genotype effects on gene expression and PGE_2_ levels in colon biospies

There was no effect of aspirin treatment or *UGT1A6* genotype on gene expression in stromal or epithelial tissue as measured by microarray. The distributions of p-values for these comparisons were no different from those under the null hypothesis and none of the estimated FDRs was less than 0.3 (data not shown).

In contrast, we found statistically significant effects of the aspirin intervention on PGE2 levels in colon tissue. PGE_2_ concentrations were approximately 40% lower after aspirin compared to placebo (0.17 ± 0.10 and 0.32 ± 0.22 ng/mg protein, respectively). The intervention effect was statistically significant (p <0.001) both with and without adjusting for age, sex, and genotype, but there was no modifying effect of genotype on the response to aspirin.

## Discussion

It is well established that colonic neoplastic tissue is epithelial in origin; however, the surrounding stroma is also believed to play an active role in this process, albeit a less well studied one [59,60]. There is evidence that changes in stromal tissue contribute to carcinogenesis and that tumorigenic epithelial cells can induce changes in stromal fibroblasts [61]. There is also increasing evidence that tumor cells actively recruit stromal cells, such as inflammatory cells, vascular cells, and fibroblasts, into the tumor [13,62]. Thus, the tissue microenvironment and communication between these two tissues play an important role in tumor-cell proliferation and in progression [15,63]. Although many factors have been identified as stromal signals [41], the pathways involved in this intercellular ‘cross-talk’ are only partly understood. Differential gene sets and biological pathway characterization to study tumorigenesis [8] and to classify molecular subtypes in colon cancer [64] have greatly contributed towards our understanding of colon cancer heterogeneity.

Here, we present, for the first time, a large-scale study of tissue-specific gene expression patterns in normal human colon from healthy individuals. Separation of epithelial and stromal fractions was confirmed through the analysis of genes known to be restricted to either stroma or epithelium from previous immunohistochemistry studies. Good corroboration was seen between gene expression and immunohistochemistry studies (Figure 2). CD44, which is expressed in the crypt base, showed higher expression in the stroma than in the epithelium. This could indicate that epithelial cells at the crypt base remained partially attached to the stromal fraction, i.e., that the cell separation primarily removes the luminal epithelial cells but did less well with the more basal cells. Equally, however, the strong expression of immune-related genes in the stroma (see Additional file 6) could suggest that CD44 expression by hematopoietic cells is the cause of higher relative expression in the stroma.

Our quantitative RT-PCR assay results suggest that the technique used to separate epithelium from stroma resulted in a relatively clean and homogeneous epithelial fraction. Specifically, we observed little to no expression of the two stromal genes in the epithelial samples (Figure 3). However, as noted, the stromal fraction expressed detectable amounts of both of the examined epithelial genes, albeit at lower levels than the epithelium.

Our results both corroborate and contrast with other studies of gene expression in the colon. Genes involved in the accumulation, biosynthesis, metabolism, and modification of fatty acids and lipids were highly represented in the epithelium (see Additional file 2), consistent with exposure of the epithelial cells to the colonic lumen and the uptake of nutrients, including short-chain fatty acids, produced by colonic bacterial fermentation of fiber and other carbohydrates. Additionally, as noted in Table 2, protein trafficking, amino acid, nucleic acid, and carbohydrate metabolism were also represented more in epithelial tissue. The prostaglandin pathway is of particular interest for colorectal carcinogenesis [65,66] as it is a main target of NSAIDs. Colorectal tumors also express high prostaglandin levels [65,66]. As shown in Additional file 3, patterns of expression of these genes were seen in both epithelium and stroma but differed between them.

Our data are also consistent with previously published data showing that colonic epithelium expresses genes associated with ion transport of Na+, Cl^−^, H^+^, K^+^, Cu^2+^ and other heavy metals, as well as genes necessary for the metabolism of xenobiotics and drugs (see Additional files 4 and 5) [9,10]. We observed that the molecular-transport genes expressed more highly in the stroma were associated with Ca^2+^ transport. This separation of ion transport function is consistent with the role of epithelial cells in water absorption and electrolyte transport and the use of Ca^2+^ signaling by cells in the stromal compartment, including hematopoietic cells [67,68].

Sugiyama et al. [11] found increased expression of genes involved in angiogenesis, invasion/metastasis in cancer stroma compared with normal stroma. Our data showed that the genes involved in invasion/metastasis, such as TIMP1 and MMP1, also show higher expression in normal stroma than in epithelium (see Additional files 6 and 7). Several of the tumor endothelial markers observed by St. Croix and colleagues [13] such as CTGF, MGP, COL5A1, VWF, and nidogen (NID1) were also more highly expressed in stroma in our study, again showing that endothelial and angiogenesis-related factors are characteristic of normal stroma.

Saaf et al. [69] reported that IL10RB and IL6 had higher expression in polarized Caco-2 epithelial cells (which mimic colonic epithelial cell differentiation). In contrast, we observed an extensive list of genes involved in inflammation and immune response in stroma but not in epithelium, thereby providing additional evidence for differences between normal intact tissue behavior and that of transformed cells in culture.

Mariadason et al. [9] reported increased expression of ECM components during Caco-2 cell differentiation. Halbleib et al. [10] also found genes encoding ECM components to be regulated during Caco-2 cell polarization. In contrast to these *in vitro* studies, however, and consistent with the inflammatory-gene data above, we found that several ECM components (collagens, laminins, fibulin, fibronectin) were specifically expressed in stroma, suggesting that, whereas the Caco-2 *in vitro* model mimics the epithelial absorptive cell lineage differentiation, those cells do not mirror the expression patterns observed in normal human colonic epithelium and stroma. This, plausibly, could result from lack of interaction between Caco-2 cells (which are derived from tumor and hence far removed from any normal cell) and a normal stromal milieu. Our results clearly suggest caution in interpreting observations from *in vitro* models of tissue development that lack the characteristics of the *in vivo* tissue microenvironment and, specifically, the complex mix of cells that characterize the normal colonic stroma.

Constitutive activation of Wnt/β-catenin signaling is an early event in the development of colorectal carcinomas in humans [55,70]. The Wnt/wingless pathway has a probable morphostatic role because of its control of epithelial-cell proliferation and differentiation along the crypt-villus axis [59]. The Wnt signaling pathway was also one of the top canonical pathways associated with the differentially expressed gene list generated from the comparison of tumor to normal cells in the work of Mojica and Hawthorn [16]. Our observation that some Wnt pathway and target genes are specific to stroma whereas others are expressed more highly in epithelium was intriguing, so we investigated the subcellular localization of the Wnt pathway genes in the stroma and epithelium by using IPA (see Additional file 9). More of the secreted proteins showed higher expression in the stroma whereas higher expression of the cytoplasmic proteins was seen in the epithelium. Our results suggest that the stroma is the major source of soluble factors related to the Wnt pathway within the colon. These data allow further dissection of the contribution that these important pathways make in establishing a healthy tissue microenvironment in the colon.

We looked at the 4 different colon cancer molecular subtypes described by Perez-Villamil et al. [64] and compared their differential gene sets characterizing each tumor subtype with our stromal and epithelial differential gene expression data. We found that, 33% of genes belonging to their cluster-2 (Immunoglobulin-related-subtype) and 51% of their cluster- 3 (High-stroma-subtype) showed higher expression in our stromal data set versus 4% and 0%, respectively, in our epithelial data set. Genes belonging to their cluster-4 (Mucinous-subtype) showed higher expression in our epithelial fraction. Of the genes that showed lowest expression in cluster-1 (low-stroma-subtype), 11 were expressed more highly in our epithelial dataset (includes metallotheioneins and carboxymetallopeptidase M) and 38 (mixture of mostly immune-system-related genes as well as some apolipoproteins) showed higher expression in our stromal data set.

Mojica and Hawthorn [16] have presented a reference data set of gene expression in normal colonic epithelial cells and compared it to tumor and matched normal colon. However, they note that adjacent normal tissue is highly heterogeneous and not always representative of normal colon cells. Our gene analysis of normal healthy colon epithelium and stroma adds considerable value when used in conjunction with their differential-gene-expression study. Furthermore, genes identified as being down-regulated in colorectal adenoma compared to normal mucosa were differentially expressed between stroma and epithelium in our study, providing additional evidence that changes during carcinogenesis occur in both epithelial and stromal tissue [71].

In this study of healthy men and women, we did not detect statistically significant differences in gene expression with aspirin treatment, as measured by microarray of normal colon tissue. However, the differences we observed in tissue PGE_2_ levels between aspirin treatment and placebo provide support for a dose and duration sufficient to elicit a response *in vivo*. Previously, we showed in the same study, that aspirin treatment did not change expression of 15-hydroxyprostaglandin dehydrogenase (15-PGDH)—the enzyme that catabolizes PGE_2_ by oxidizing its 15(s)-hydroxy group—suggesting that aspirin does not affect PGE_2_ levels through modulation of 15-PGDH [72]. Given the observation that, in normal colonic tissue, Cox-1 contributes to the majority of PGE_2_ production [73], inactivation of Cox-1 by aspirin’s acetylation of strategically located serine residues [74] is likely a primary mechanism of reduced PGE_2_. In humans, aspirin doses as low as 81 mg/d were sufficient to significantly lower PGE_2_ in colorectal mucosa [75]. Similarly, Bousserouel et al. [76] showed that aspirin supplementation reduced both normal mucosal PGE_2_ concentrations and aberrant crypt foci by approximately 50% in rats treated with azoxymethane.

Several technical and logistical aspects of the study may have made it more difficult to detect subtle changes in gene expression in response to aspirin. The Fleet Phospho-soda Oral Saline Laxative may have changed gene expression in the colon cells to a greater degree such that any effects of aspirin could not be detected. We might have expected this to have less effect on the underlying stromal component; however, no effect was seen in this tissue sub-type either. Further, we relied on 2-cycle amplification, which may have contributed more variation in transcript levels and reduced our capacity to detect any aspirin-related differences. A limitation of this study is the selection of participants based solely on their UGT1A6*2 genotypes with no screening or selection for polymorphisms in other genes involved in aspirin metabolism, such as CYP2C9 and ACSM2 [17,22,77]. We also selected only younger healthy adults and the study was not designed for long-term follow-up. However, the American Cancer Society recently reported that, although CRC incidence rates have declined by 3.7% per year from 2006–2010 among adults 50 years and older, they have increased by 1.8% per year among adults younger than 50 years. This observation suggests that a large population-based study in younger adults may be warranted.

## Conclusions

We conducted a comprehensive study of differential gene expression between normal human colonic epithelium and stroma from healthy individuals. We have identified the genes uniquely and reproducibly expressed in each tissue type and have analyzed the biologic processes they represent. The wide differences in expression between stroma and epithelium suggest that future study of gene expression in colonic epithelium may benefit from separation of the tissue so as to focus the analysis on a specific tissue type.

## Additional files

Additional file 1:
**List of 4916 statistically significant genes and their expression values.** There are 4 samples from each person designated A1-A4 (person 1); A5-A6 (person 2); B1-B4 (person 3) etc. to W1-W4 (person 44). Within each person, odd numbers (1 & 3) are epithelial tissue and even numbers (2 & 4) are stromal tissue. The epithelial and stroma from each intervention period are paired together (e.g., A1 & A2 are from period 1; A3 & A4 are from period 2). *Note: The type of intervention linked to each period is masked. Please contact the study authors directly if you would like access to this information*. Microarray data are publicly available in Gene Expression Omnibus (GEO). Additional file 2:
**Expression of lipid metabolism genes in colonic epithelium and stroma.** Top 25 genes involved in lipid metabolism (by p-value) that showed higher expression in epithelial tissue compared to the corresponding stromal tissue. Red indicates over-expressed genes and green indicates under-expressed genes; and the expression level is proportional to the brightness of the color (see color bar). Black indicates no difference in expression level between the two tissue types and lighter coloring indicates lower overall expression. Additional file 3:
**Expression of prostaglandin pathway genes in colonic epithelium and stroma.** Heatmap of relative expression of selected statistically significant genes involved in the prostaglandin pathway. Relative expression levels ranged from bright green (lowest) to bright red (highest). Black indicates no difference in expression level between the two tissue types and lighter coloring indicates lower overall expression. Additional file 4:
**Expression of molecular transport genes in colonic epithelium and stroma.** The heatmap of relative expression of selected statistically significant genes involved in molecular transport showed higher expression in epithelium compared to stroma. Heatmap relative expression levels ranged from bright green (lowest) to bright red (highest). Black indicates no difference in expression level between the two tissue types and lighter coloring indicates lower overall expression. Additional file 5:
**Expression of genes in the drug and xenobiotic metabolism pathway in colonic epithelium and stroma.** The heatmap of relative expression of selected statistically significant drug and xenobiotic metabolism genes demonstrated higher expression in epithelium for the majority of genes. Relative expression levels ranged from bright green (lowest) to bright red (highest). Black indicates no difference in expression level between the two tissue types and lighter coloring indicates lower overall expression. Additional file 6:
**Stroma-specific expression of inflammation and immune response genes in biopsy samples of human colon.** The heatmap of selected statistically significant genes involved in inflammation and immune response showed the strong stroma-specific expression of immune-related genes. Relative expression levels ranged from bright green (lowest) to bright red (highest). Black indicates no difference in expression level between the two tissue types and lighter coloring indicates lower overall expression. Additional file 7:
**Expression of extracellular matrix genes in colonic stroma and epithelium.** Twenty-five matrix modeling and extracellular matrix genes (10 epithelial genes and 15 stromal genes) with different levels of expression in epithelium compared with stroma. (Relative expression levels range from bright green (lowest) to bright red (highest). Black indicates no difference in expression level between the two tissue types and lighter coloring indicates lower overall expression. Additional file 8:
**Wnt-Tcf4 pathway gene expression in colonic epithelium and stroma.** Heatmap of all statistically significant Wnt target and pathway genes identified from the Wnt homepage and IPA and Genespring databases. Two distinct tissue-specific expression patterns were observed – see Discussion and Additional file 9. Relative expression levels ranged from bright green (lowest) to bright red (highest). Black indicates no difference in expression level between the two tissue types and lighter coloring indicates lower overall expression. Additional file 9:
**Cellular location of the products of 42 Wnt genes that were preferentially expressed by stroma or epithelium.** Ingenuity Pathway analysis was used to analyze the cellular location of differentially expressed genes. Genes associated with production of secreted proteins (e.g., DKK3, Wnt5A and Wnt5B) were expressed preferentially in stroma. Genes associated with production of cytoplasmic proteins were expressed preferentially in epithelium.

## Abbreviations

AGT: Angiotensinogen
COX: Cyclooxygenase
CRC: Colorectal cancer
ECM: Extracellular matrix
FDR: False discovery rate
GO: Gene Ontology
IPA: Ingenuity pathway analysis
LOH: Loss of heterozygosity
MHC: Major histocompatibility complex
NSAID: Non-steroidal anti-inflammatory drug
PGDH: Prostaglandin dehydrogenase
PGE_2_: Prostaglandin E2
UGT: UDP-glucuronosyltransferase
