# Supplementary figures and images for: Tissue-specific patterns of gene expression in the epithelium and stroma of normal colon in healthy individuals in an aspirin intervention trial
Source: BMC Med Genet. 2015 Mar 24;16:18. doi: 10.1186/s12881-015-0161-6 (PMC4422425; doi:10.1186/s12881-015-0161-6)

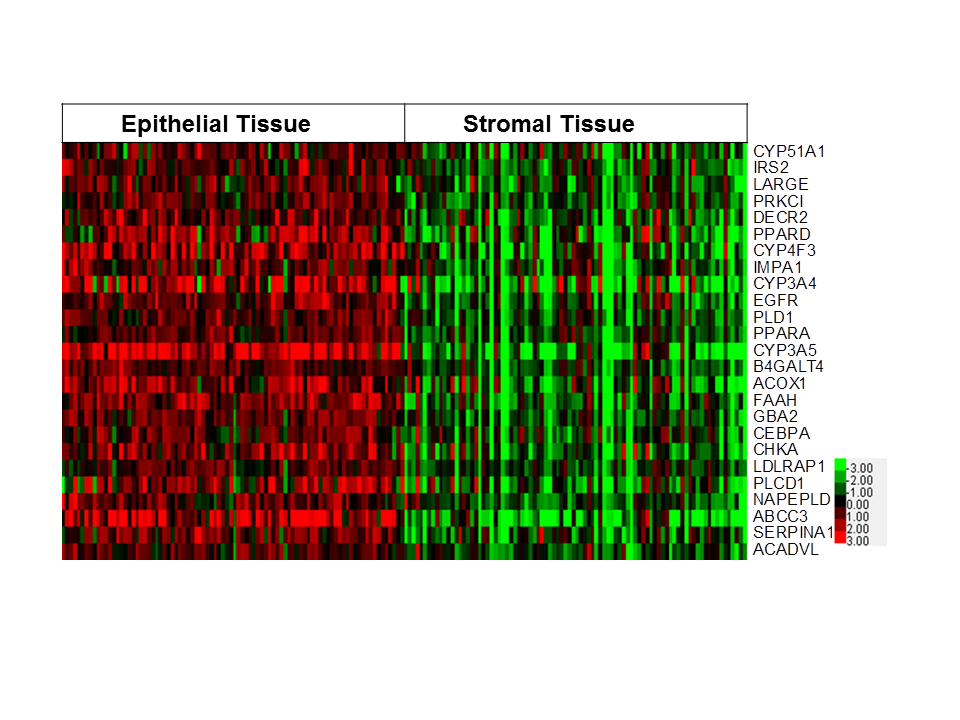

Supplement: Additional file 2: — Expression of lipid metabolism genes in colonic epithelium and stroma. Top 25 genes involved in lipid metabolism (by p-value) that showed higher expression in epithelial tissue compared to the corresponding stromal tissue. Red indicates over-expressed genes and green indicates under-expressed genes; and the expression level is proportional to the brightness of the color (see color bar). Black indicates no difference in expression level between the two tissue types and lighter coloring indicates lower overall expression. [file 12881_2015_161_MOESM2_ESM.png]

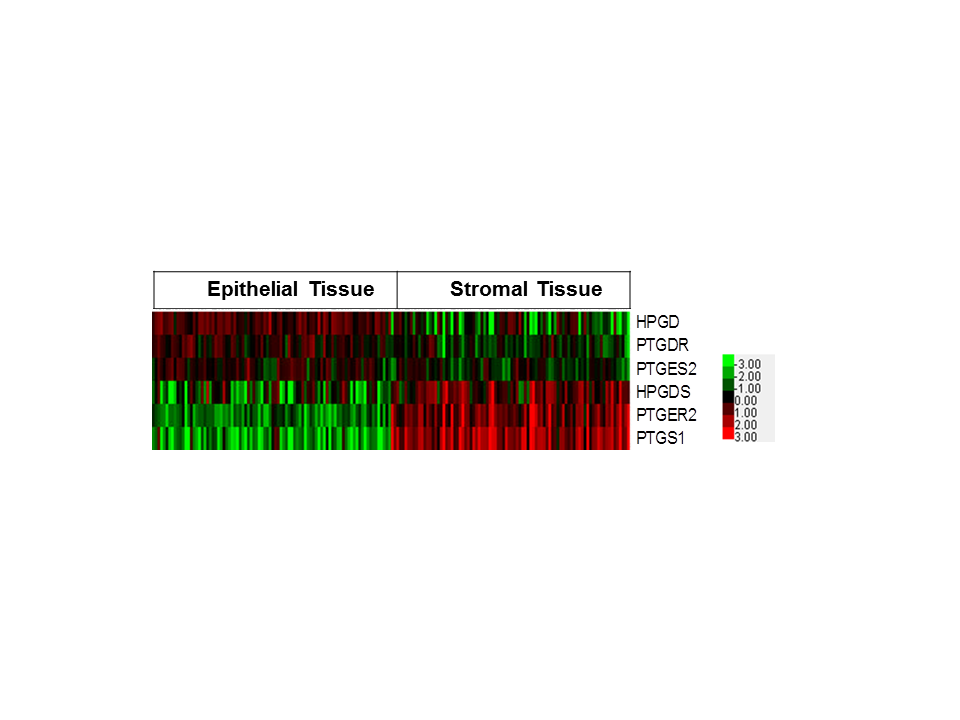

Supplement: Additional file 3: — Expression of prostaglandin pathway genes in colonic epithelium and stroma. Heatmap of relative expression of selected statistically significant genes involved in the prostaglandin pathway. Relative expression levels ranged from bright green (lowest) to bright red (highest). Black indicates no difference in expression level between the two tissue types and lighter coloring indicates lower overall expression. [file 12881_2015_161_MOESM3_ESM.png]

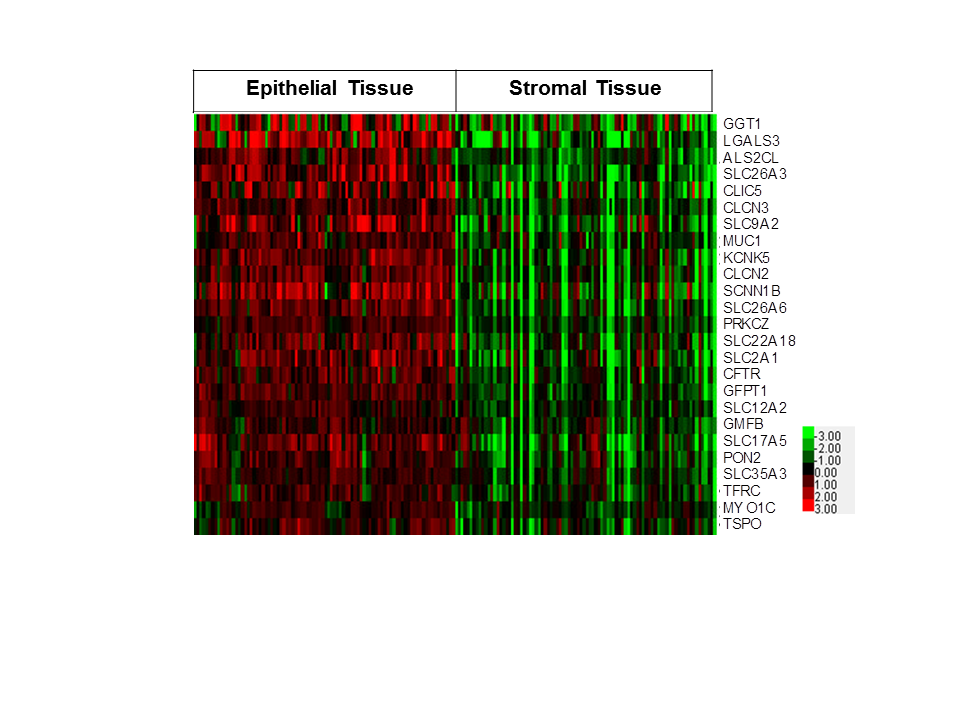

Supplement: Additional file 4: — Expression of molecular transport genes in colonic epithelium and stroma. The heatmap of relative expression of selected statistically significant genes involved in molecular transport showed higher expression in epithelium compared to stroma. Heatmap relative expression levels ranged from bright green (lowest) to bright red (highest). Black indicates no difference in expression level between the two tissue types and lighter coloring indicates lower overall expression. [file 12881_2015_161_MOESM4_ESM.png]

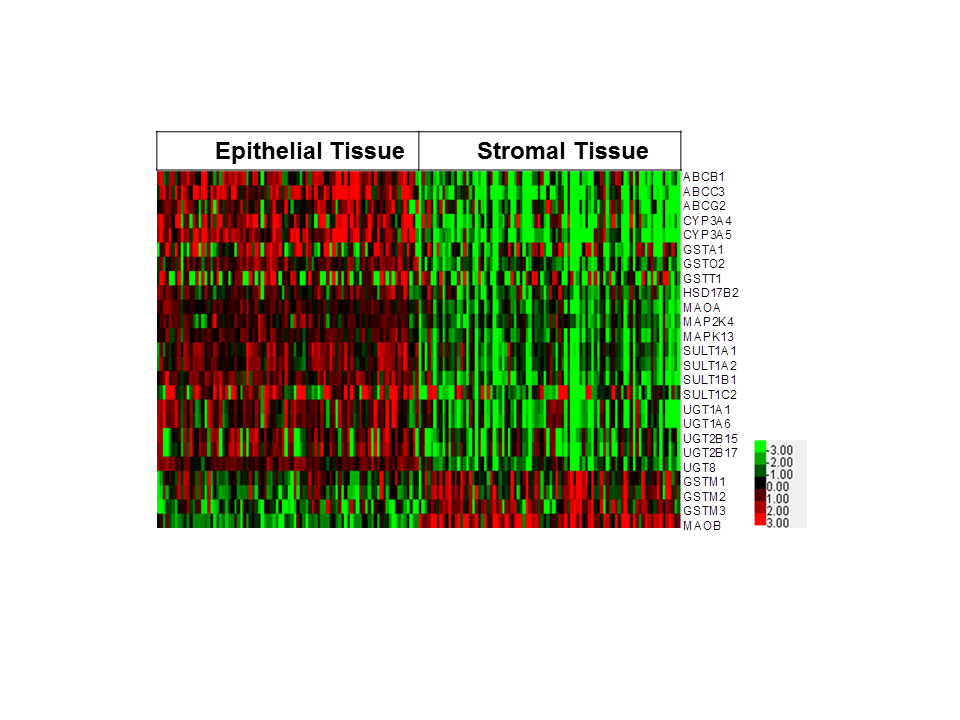

Supplement: Additional file 5: — Expression of genes in the drug and xenobiotic metabolism pathway in colonic epithelium and stroma. The heatmap of relative expression of selected statistically significant drug and xenobiotic metabolism genes demonstrated higher expression in epithelium for the majority of genes. Relative expression levels ranged from bright green (lowest) to bright red (highest). Black indicates no difference in expression level between the two tissue types and lighter coloring indicates lower overall expression. [file 12881_2015_161_MOESM5_ESM.png]

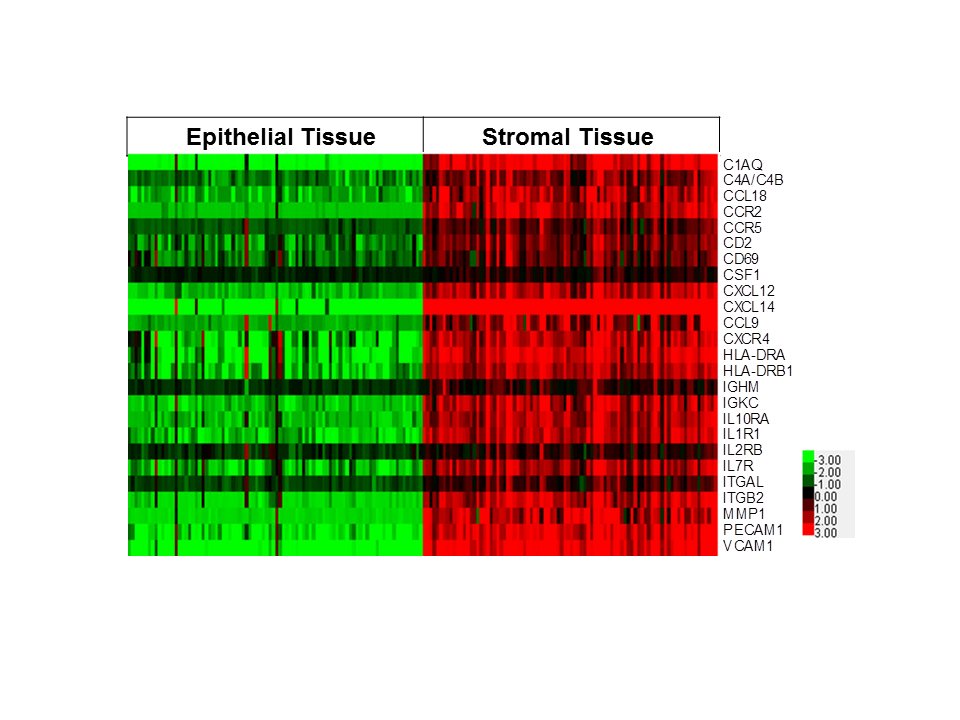

Supplement: Additional file 6: — Stroma-specific expression of inflammation and immune response genes in biopsy samples of human colon. The heatmap of selected statistically significant genes involved in inflammation and immune response showed the strong stroma-specific expression of immune-related genes. Relative expression levels ranged from bright green (lowest) to bright red (highest). Black indicates no difference in expression level between the two tissue types and lighter coloring indicates lower overall expression. [file 12881_2015_161_MOESM6_ESM.png]

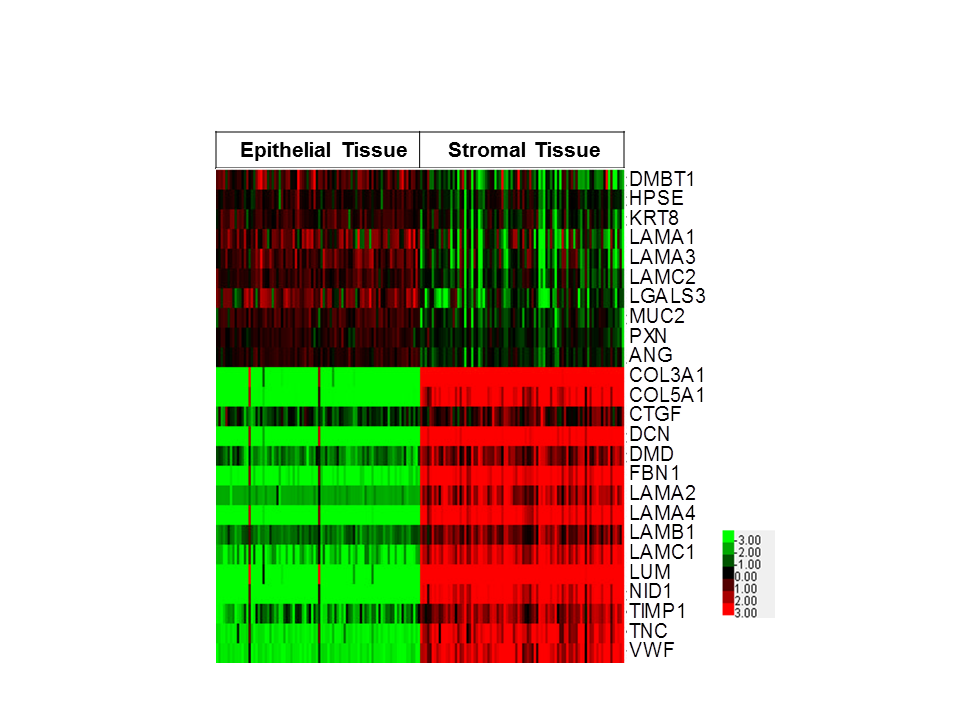

Supplement: Additional file 7: — Expression of extracellular matrix genes in colonic stroma and epithelium. Twenty-five matrix modeling and extracellular matrix genes (10 epithelial genes and 15 stromal genes) with different levels of expression in epithelium compared with stroma. (Relative expression levels range from bright green (lowest) to bright red (highest). Black indicates no difference in expression level between the two tissue types and lighter coloring indicates lower overall expression. [file 12881_2015_161_MOESM7_ESM.png]

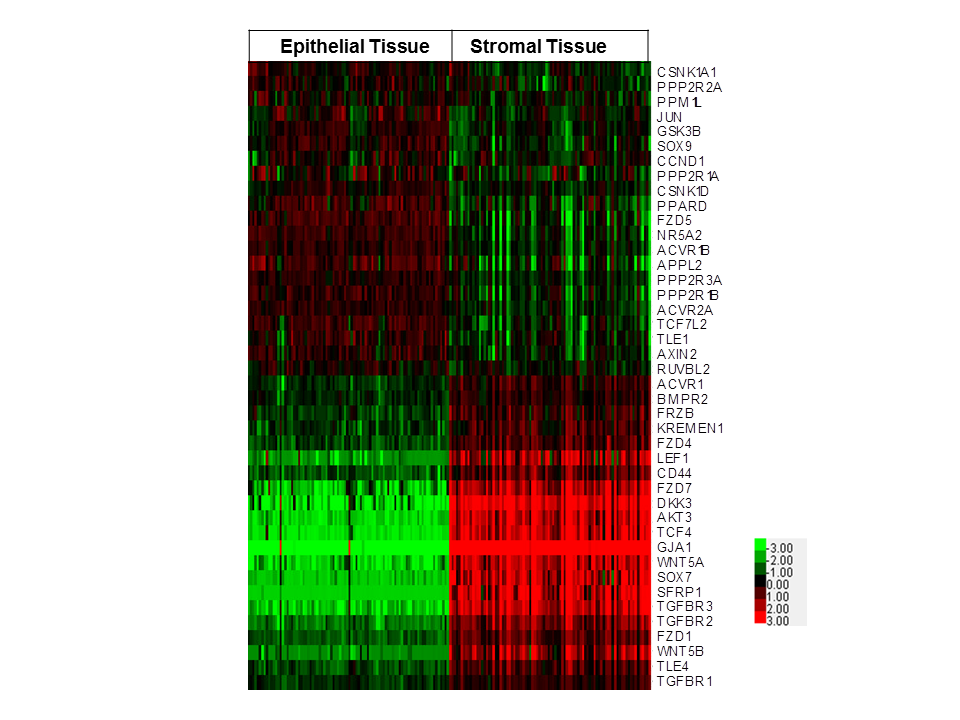

Supplement: Additional file 8: — Wnt-Tcf4 pathway gene expression in colonic epithelium and stroma. Heatmap of all statistically significant Wnt target and pathway genes identified from the Wnt homepage and IPA and Genespring databases. Two distinct tissue-specific expression patterns were observed – see Discussion and Additional file 9. Relative expression levels ranged from bright green (lowest) to bright red (highest). Black indicates no difference in expression level between the two tissue types and lighter coloring indicates lower overall expression. [file 12881_2015_161_MOESM8_ESM.png]

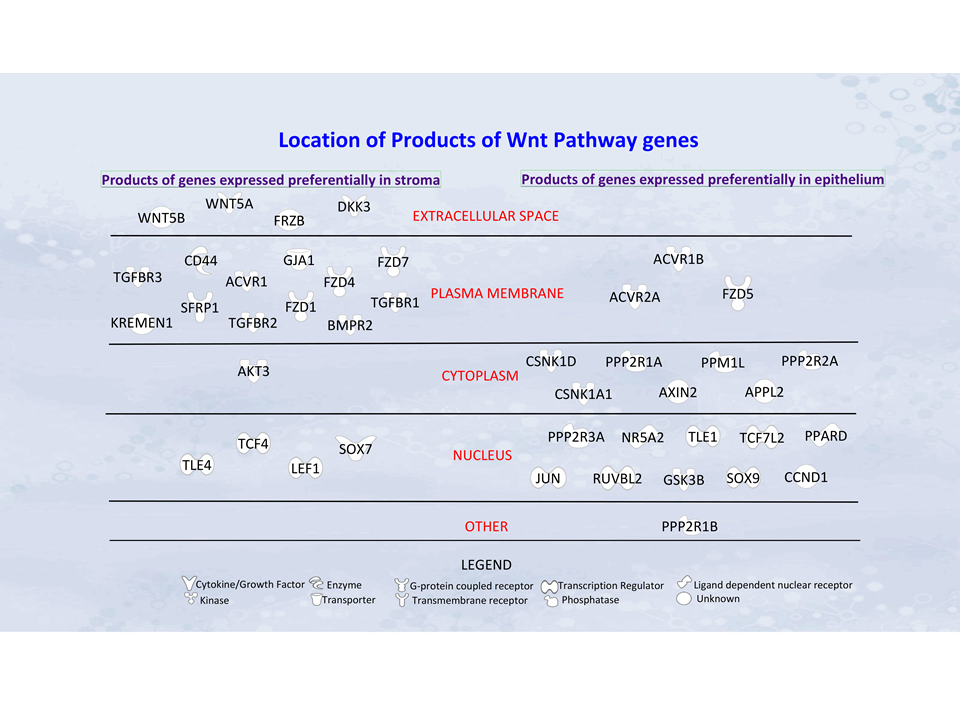

Supplement: Additional file 9: — Cellular location of the products of 42 Wnt genes that were preferentially expressed by stroma or epithelium. Ingenuity Pathway analysis was used to analyze the cellular location of differentially expressed genes. Genes associated with production of secreted proteins (e.g., DKK3, Wnt5A and Wnt5B) were expressed preferentially in stroma. Genes associated with production of cytoplasmic proteins were expressed preferentially in epithelium. [file 12881_2015_161_MOESM9_ESM.png]
